# Altered activation patterns of the sensory-motor cortex in patients with knee osteoarthritis during knee isokinetic movement at different speeds

**DOI:** 10.3389/fbioe.2024.1444731

**Published:** 2024-08-21

**Authors:** Kun Yang, Yuwu Ding, Lixi Chu, Changfeng Cheng, Xiaoming Yu, Haichen Xu, Ying Tao, Tiantian Liu, Lei Yin, Xubo Wu, Bingli Liu, Liming Jiang

**Affiliations:** ^1^ Department of Rehabilitation, Seventh People’s Hospital of Shanghai University of Traditional Chinese Medicine, Shanghai, China; ^2^ Department of Traditional Chinese Medicine, Shanghai Puxing Community Healthcare Center, Shanghai, China; ^3^ Department of Orthopedics and Traumatology, Seventh People’s Hospital of Shanghai University of Traditional Chinese Medicine, Shanghai, China

**Keywords:** knee osteoarthritis, cortex activation, functional near-infrared spectroscopy, isokinetic, pain

## Abstract

**Background:**

Abnormal brain activation patterns in patients with knee osteoarthritis (KOA) at rest have been revealed, but it is unclear how brain activation patterns change during movement. This study aimed to investigate the alterations in brain activation patterns in KOA patients during knee isokinetic movement, and the correlation between cortical activity changes and pain severity and dysfunction.

**Methods:**

Eighteen patients with KOA and 18 healthy controls (HC) were recruited, and to performed the knee isokinetic test with three speeds. Functional near-infrared spectroscopy (fNIRS) was used to detect the cerebral cortex hemodynamics changes of primary somatosensory (S1), primary motor (M1) and somatosensory association cortex (SAC) in the region of interest (ROI) during movement. Then, we evaluated potential correlations between M1, S1 and SAC values and Western Ontario and McMaster Universities Osteoarthritis Index (WOMAC) and visual analog scale (VAS) scores.

**Results:**

The results showed that peak torque of knee extension in KOA patients was significantly smaller than that in HC. For HC, unilateral knee movement activated bilateral ROIs. The contralateral activation was dominant, showing the phenomenon of high contralateral activation. For KOA patients, there were no statistical difference in the activation level between the left and right of the cerebral cortex, with both sides showing lower activation levels compared to HC. Further analysis found that the contralateral M1, S1, and SAC of the affected knee in KOA patients were significantly lower than those in HC, while no difference was found on the ipsilateral side. Moreover, during isokinetic movement at 180°/s, VAS score in KOA patients was negatively correlated with the activation level of the contralateral S1 and M1 values, and WOMAC was negatively correlated with the activation level of the contralateral M1 value.

**Conclusion:**

Contralateral activation of the sensorimotor cortex exists during unilateral knee movement, but in KOA patients, this contralateral cortical activation is suppressed. Furthermore, the clinical pain and dysfunction in KOA patients are associated with activation levels of specific brain regions. These findings can provide a better understanding of KOA brain science and are expected to contribute to the development of central intervention for the disease.

## 1 Introduction

Knee osteoarthritis (KOA) is a chronic degenerative bone and joint disease common in middle-aged and older adults (Glyn-Jones et al., 2015). It often leads to persistent pain and functional changes, which severely restricts daily activities and movements (Mantana et al., 2020). According to the World Health Organization (WHO), KOA is recognized as one of the most prevalent disabling conditions worldwide (Cross et al., 2014). It has been reported that, more than 1/3 of the elderly suffer from KOA, and accounts for 2.2% of overall years impacted by disability (Cross et al., 2014; Leigh F et al., 2021). Due to the aging of the global population, the incidence of KOA is expected to continue to increase, with adverse social and economic consequences (Ian J et al., 2017).

Previous studies showed that although KOA is typically considered a peripheral joint disease, it has been demonstrated that the occurrence and development of KOA are related to abnormalities in the central nervous system (CNS) (Enrique et al., 2017; Ye et al., 2023). However, less is known about the effects of KOA on CNS changes (Mansfield et al., 2022). It has been found that as even after KOA patients receive traditional rehabilitation management, abnormal activation patterns and changes of CNS still persist (Özgül et al., 2021). Several studies have used different methods to observe the abnormal CNS changes caused by KOA (Shanahan et al., 2015; Cheng et al., 2022; Kang et al., 2022). Jerin discovered that abnormalities in electroencephalography (EEG) patterns at sensory discriminative, and descending inhibitory cortical areas were exhibited in KOA patients (Jerin et al., 2023). Cheng and Kang found by magnetic resonance imaging (MRI) that compared with healthy controls, KOA patients had decreased motor cortex (gray matter and white matter) volume and altered resting-state brain networks, and the degree of change was correlated with disease severity (Cheng et al., 2022; Kang et al., 2022). Shanahan et al. found by MRI that KOA patients experienced significant changes in the motor cortex activation sites when performing visually guided force-matched isometric contraction task, and these changes were associated with modified motor function and behavior (Shanahan et al., 2015). The authors believe that this may be an important reason for the poor motor performance of KOA patients (Shanahan et al., 2015). This partly confirms that KOA patients have motor deficits associated with decreased neural activation. However, these studies were conducted in a resting state and cannot detect the brain activation changes during real movement tasks, which is important for understanding of KOA. The patterns of brain activation observed during movement can reflect the dysfunction of patients in daily life activities, and reveal the potential obstacles of patients in motor control and coordination (Andres M et al., 2017). Furthermore, it is unclear brain activation patterns relate to function and pain during movement. Especially the function, which is more meaningful reflected in movement tasks. Consequently, an in-depth understanding of brain activation patterns during movement is crucial for advancing the fields of neuroscience and neurorehabilitation related to KOA.

In recent years, advances in functional near-infrared spectroscopy (fNIRS) provides convenience for the study of brain activation patterns during movement (Stephane and Pierre, 2018; Cristina et al., 2020; Alka et al., 2021). The brain activation pattern is defined as the movement task-related activation representations in the specific area of the brain cerebral cortex (Eliassen et al., 2001). The rationale for fNIRS measured brain activation pattern is based on the concept that neural activation in response to external stimuli or movement tasks results in increased hemodynamic in the activated area. Borot L used fNIRS to observe the brain activation patterns during limb movement in healthy participants and found that fNIRS measurements were highly reliable and valid compared with fMRI measurements (Borot et al., 2018). For the application of fNIRS in KOA, Özgül et al. (2021) found that movement improved clinical assessments of pain severity after 6 weeks of exercise training in KOA patients, accompanied by changes in prefrontal cortex activation monitored by fNIRS. Pollonini et al. (2020) used fNIRS research to find that after the treatment of transcranial direct current stimulation (tDCS), the excitability of related functional brain regions in patients with KOA significantly increased. The above studies observed changes of cortical hemodynamic in brain regions of KOA patients through peripheral and central interventions respectively, but these were only monitored by fNIRS in a resting state and lacked healthy controls.

It is unclear how the brain activation patterns in KOA change during real movement tasks, especially including the hemodynamic changes of primary somatosensory (S1), primary motor (M1) and somatosensory association cortex (SAC). In addition, there is a lack of research comparing cerebral hemodynamic changes in KOA patients and healthy controls during movement, and whether these changes can serve as reliable objective measurements of pain and functional changes in patients with KOA. Therefore, this study used fNIRS to explore the changes of brain activation patterns in KOA patients and healthy subjects during movement, and the correlation between these changes and pain severity and dysfunction in patients with KOA, which will provide evidence and potential targets for brain activation changes for subsequent interventions, and open a new perspective for the rehabilitation of musculoskeletal chronic diseases. We hypothesized that KOA patients had different brain activation patterns during knee isokinetic movement compared to the healthy controls, and the clinical pain and dysfunction in KOA patients were associated with activation levels in specific brain regions.

## 2 Materials and methods

### 2.1 Study design

A biomechanical-fNIRS cross-section study was conducted to examine the brain activation patterns and knee torque in the KOA patients during knee isokinetic movement at three speeds (60, 120, and 180°/s) as compared to the healthy controls (HC), and to identify the changes of brain activation patterns during movement, and the correlation between these changes and pain severity and dysfunction in KOA patients.

### 2.2 Sample size calculation

The determination of the sample size was accomplished by conducting *a priori* power analysis using the G*Power software (version 3.1.9.7). A previous study reported that the patients with KOA showed reduced maximal knee extensor isometric torque compared with the healthy controls (mean value: 1.35 vs. 1.87 Nm/kg) (Carlos et al., 2021). The present study calculated the effect size was 1.14 based on this result, an α error of 0.05 and β of 0.95 (power level of 0.95), allocation ratio 1, and determined that a minimum of 36 participants were required to ensure an appropriate group size for the study, resulting in a sample size of 18 individuals per group. This sample size was deemed sufficient to yield reliable and valid results for the present study.

### 2.3 Participants

Eighteen patients with KOA (63.88 ± 6.50 years) and eighteen demographically similar HC (61.22 ± 5.86 years) were included in this study. KOA patients were recruited from Shanghai Seventh People’s Hospital and nearby community centers through flyers, posters, and referrals from orthopedists and physiotherapists between February and March 2023. The inclusion criteria were as follows: 1) diagnosis of KOA according to the clinical classification criteria of American College of Rheumatology; 2) age range 50–75 years old; 3) having Kellgren-Lawrence grade Ⅱ and Ⅲ; 4) knee pain score (At least in the past 3 months) ≥ 3 on a 10-point scale; 5) knee symptoms and imaging manifestations are only on the right side; 6) signing informed consent form. The exclusion criteria included the following: 1) patients with other joint diseases such as rheumatoid arthritis or other inflammatory disease; 2) patients with a history of knee injury (e.g., meniscus, ligament injury, surgery); 3) patients with cardiovascular and cerebrovascular diseases, neurological or psychiatric disorder; 4) consuming medications that alter the excitability of the cerebral cortex (e.g., sedatives, stimulants). Healthy controls were recruited from communities around the hospital through flyers and posters. All participants were required to have the right dominant lower limb, which was identified by preference for kicking. Furthermore, they did not include participants who may affect exercise and brain activity (history of neurological, physical, or psychiatric illness).

### 2.4 Experimental procedure

The experiment was conducted in the rehabilitation evaluation room of the hospital, and the test environment was kept quiet throughout the procedure. All participants’ height, weight and other demographic characteristics were collected before the test. KOA patients were also evaluated with clinical measures by an experienced physiotherapist. Clinical pain was evaluated using the Visual Analog Scale (VAS) for pain, a measure of pain perception rated on a scale of 0–10 (Myles et al., 2017). KOA functional symptoms were assessed using the Western Ontario and McMaster Universities Osteoarthritis Index (WOMAC), an index specifically designed for OA ranging from 0 to 96 (Seror et al., 2007).

**FIGURE 1:**
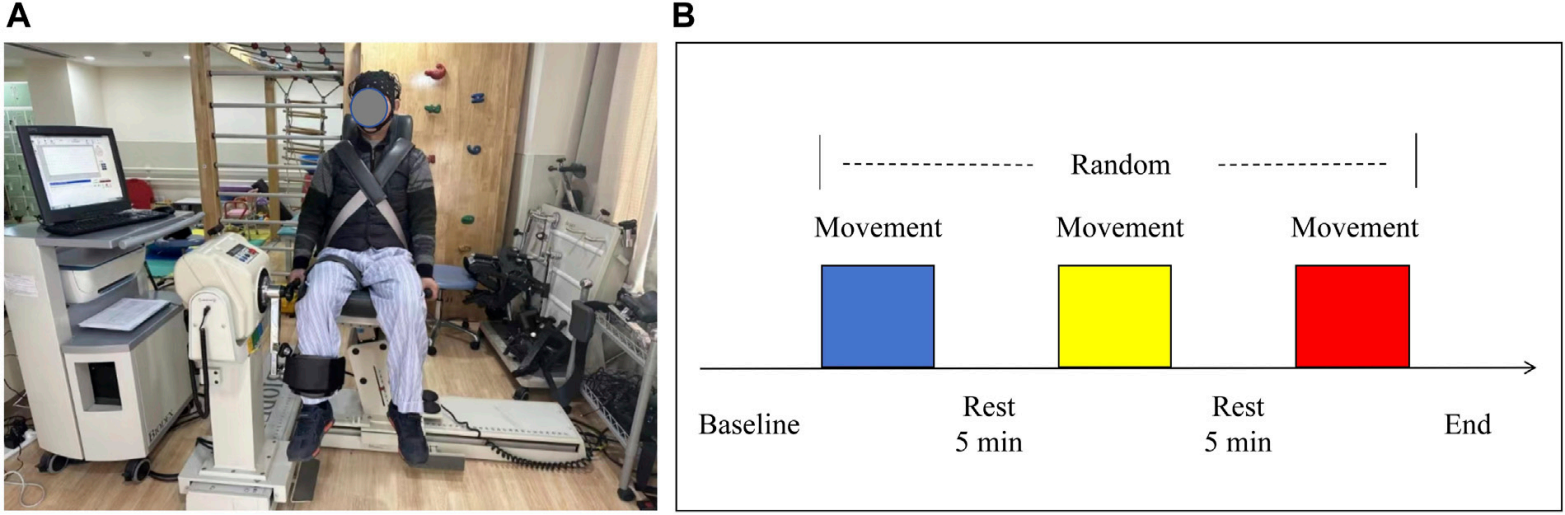
**(A)** Experimental setup and **(B)** schematic diagram of the flow for each testing session. Three colors randomly represent the isokinetic movement at 60°/s, 120°/s, and 180°/s speeds.

Before the formal test, the participants underwent two familiarization sessions to ensure they were proficient in using the technology. This practice was essential to guarantee that they were comfortable and able to perform the tasks correctly. Participants were instructed to sit on a biodex isokinetic dynamometer (System 4, Biodex Inc., Shirley, NY, United States) after wearing fNIRS device. Fasten fixation straps around the shoulders, waist and distal thigh of the test leg to increase safety and prevent any unnecessary movement during the test. The test side of all participants was on the right side. The axis of the dynamometer is positioned in alignment with the centre of the lateral femoral condyle. The lever arm was then adjusted to match the length of the leg being tested, with resistance applied to the front of the ankle. Before each trial, each participant was asked to remain in a resting state, sitting quietly for 5 min and avoiding random movements and speech to minimize the brain hemodynamic response generated by their activity. During the test, the participants completed three sets of angular velocity tests in random order: low (60°/s), medium (120°/s) and high (180°/s). The pattern was CONCENTRIC/CONCENTRIC, and each set was repeated 3 times. During each testing session, participants actively completed the flexion and extension of the knee at a constant angular speed, and exerted the maximum muscle strength as possible. Took a 5 min break between each set of tests, which allowed the fNIRS spectral signal for each test to be returned to the baseline. Each test was synchronized with a marker to keep the duration of each test consistent with the duration recorded by fNIRS. As shown in Figure 1. The isokinetic tester records the participants’ peak torque data at different angular speeds in real time.

### 2.5 fNIRS data acquisition and preprocessing

**FIGURE 2:**
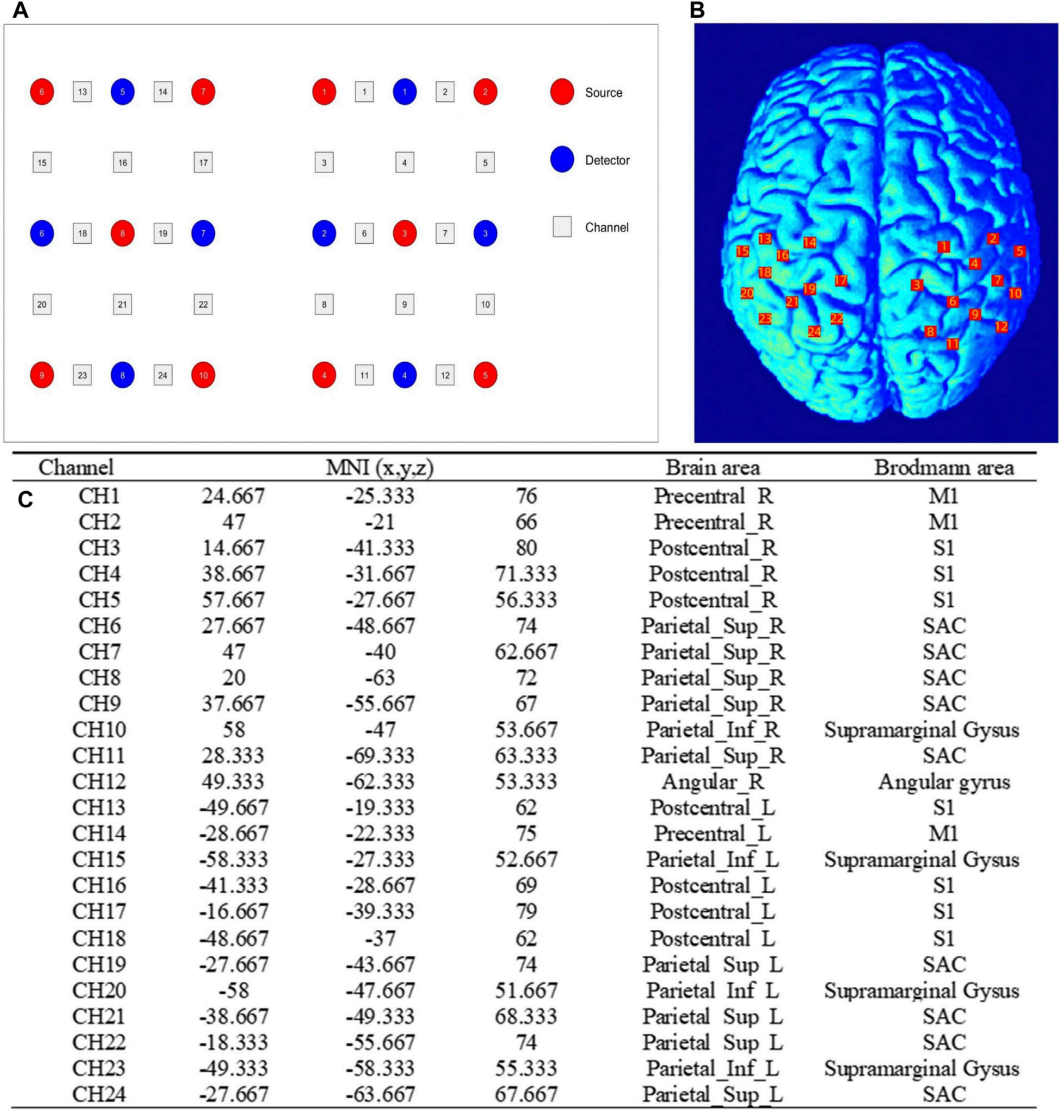
Channel assignment information for ROI. **(A)** the positions of optical emitters (red markers) and detectors (blue markers) in the fNIRS channel; **(B)** and schematic representation of fNIRS channel alignment with ROI. ROI: regions of interest; **(C)** the table related to the channel MNI coordinate, brain and Brodmann area correspondences. MNI, Montreal Neurological Institute; M1, primary motor; S1, primary somatosensory; SAC, somatosensory association cortex.

The fNIRS data was collected while the participants performed the above three sets of isokinetic movements. In order to evaluate the changes of brain activity during movement, a multi-channel wireless portable fNIRS system Brite24 system (Artinis Medical Systems, Netherlands) was used to acquire fNIRS signals from cerebral regions, making the measurement of oxyhemoglobin (HbO) and deoxyhemoglobin (HbR) possible. HbO and HbR are indirect indicators of neural activity. The changes in their concentrations reflect the local hemodynamic responses, which are closely coupled with the underlying neuronal activity. The light source of the system generates near-infrared light with two wavelengths of 670 and 850 nm, with a sampling rate of 10 Hz. Each sensor of the instrument consists of a light emitting diode and a detector photodiode, with an inter-probe distance of 30 mm. There were 10 light sources and 8 detectors in total, which constitute 24 channels. According to the location of the fNIRS channel recorded digitally in 3D, the regions covered by the probe include six regions of interest (ROI) in the bilateral brain: primary somatosensory (S1), primary motor (M1) and somatosensory association cortex (SAC). Figure 2; Table 1 shows the channel assignment information for ROI.

**TABLE 1:** Channels assigned to regions of interest.

Regions of interest	Left hemisphere	Right hemisphere
M1	14	1,2
S1	13,16,17,18	3,4,5
SAC	19,21,22,24	6,7,8,9,11

M1, primary motor cortex; S1, primary somatosensory cortex; SAC, somatosensory association cortex.

It was important to ensure that the position of the electrode Cz on the scalp surface was accurately located for research purposes. This was done by measuring the intersection from the nasal root to the anterior occipital protuberance line and from the left ear to the right ear anterior fossa line. This helps to ensure consistency in the placement of the electrode cap on the subjects when they are wearing it. Additionally, visually checking the alignment along the midsagittal plane further confirmed the accuracy of the electrode Cz position.

Raw data were extracted from Oxysoft software (v3.0.95, Artinis Medical Systems). After manual inspection and deletion of poor-quality channels, Matlab scripts from the Homer3 toolbox was used for data preprocessing (Theodore et al., 2009). The initial step involved converting the original light intensity data into optical density data by employing the hmrIntensity2OD function. To ensure data accuracy, a combination method of moving standard deviation, spline interpolation, and wavelet artefact correction function (SDThresh = 20, AMPThresh = 0.5, tMotion = 0.5 s, tMask = 2 s, p = 0.99, and iqr = 0.1) were employed to eliminate potential head motion artefacts. The corrected signal was filtered by 0.01–0.2 Hz bandpass filter (zero phase, fifth-order Butterworth filter) to eliminate physiological noise like respiration, heartbeat, and low-frequency signal drift. Finally, the corrected optical density data were converted into relative hemoglobin concentration changes in HbO and HbR by using the modified Beer-Lambert law (Kocsis et al., 2006). Since studies have shown that HbO concentration has superior signal-to-noise ratio, greater repeatability and stability over time, and a higher correlation with blood oxygen level dependent signals observed by fMRI, we used HbO concentration for follow-up analysis (Gary et al., 2002; Li. et al., 2023).

### 2.6 Statistical analysis

SPSS 21.0 software (IBM Corporation, Armonk, NY) was used for statistical analysis of results. The frequencies of count data were calculated in statistical descriptions. Data of measured data were described as mean ± standard deviation. Normality of the data was assessed with Shapiro-Wilk test. Demographic characteristics between groups were compared using independent sample t-test or Chi-square t-test. The differences in activation between left and right brain regions were analyzed by paired t-test. To assess whether brain activation differed between groups (KOA and HC) at different speeds (60°/s, 120°/s, and 180°/s), two-way repeated-measures ANOVA or non-parametric test (when normality was not met) was performed for each brain region. In addition, Pearson’s correlations were used to determine the association between changes in brain activity and VAS and WOMAC scores. Pearson’s correlations were graded as low (*r* < 0.30), moderate (0.30 < *r* < 0.60), and high (*r* > 0.60) (Monticone et al., 2018). The significance level was set at *P* < 0.05 for all analyses.

## 3 Results

### 3.1 Demographics and characteristics of the population

All 36 participants completed the study. The demographics and characteristics of the KOA patients and HC are presented in Table 2. There were no significant differences between the two groups in terms of age, gender, height, weight and BMI (*P* > 0.05). The mean VAS and WOMAC scores of patients with KOA were 4.50 ± 0.98 and 46.44 ± 5.75, respectively.

**TABLE 2:** Demographics and characteristics of the KOA patients and HC.

Characteristic	KOA (n = 18)	HC (n = 18)	t/x^2^	*P*-value
Age (years)	63.88 ± 6.50	61.22 ± 5.86	1.291	0.205
Gender (male/female)	14/4	13/5	0.148	0.700
Height (m)	1.63 ± 0.06	1.62 ± 0.06	0.332	0.742
Weight (kg)	66.91 ± 7.54	64.64 ± 8.97	0.823	0.416
BMI (kg/m^2^)	25.25 ± 2.03	24.50 ± 2.50	1.002	0.323
VAS	4.39 ± 1.03			
WOMAC	46.44 ± 5.75			

KOA, knee osteoarthritis; HC, healthy controls; BMI, body mass index; VAS, visual analog scale; WOMAC, Western Ontario and McMaster universities osteoarthritis index.

### 3.2 Isokinetic peak torque of knee extension between KOA patients and HC

Table 3 shows peak torque of knee extension of KOA patients and HC at the different speeds. There was no significant interaction effect between group and speed for peak torque (*P* = 0.186). A significant main effect of group was found for peak torque (*P* = 0.005). Compared to the HC, KOA patients had significantly smaller peak torque at all speeds. However, there were no significant speed effects for peak torque (*P* = 0.157).

**TABLE 3:** Isokinetic peak torque of knee extension of KOA patients and HC at the different speeds.

	60°/s	120°/s	180°/s	F	*P*
KOA	21.58 ± 9.34	21.31 ± 11.67	21.59 ± 10.05	0.018	0.982
HC	38.77 ± 22.24	34.06 ± 19.25	33.72 ± 16.01	2.754	0.077
F	9.646	6.094	7.820		
*P*	0.004	0.018	0.008		

KOA, knee osteoarthritis; HC, healthy controls.

### 3.3 Bilateral differences in cerebral cortex activation

**FIGURE 3:**
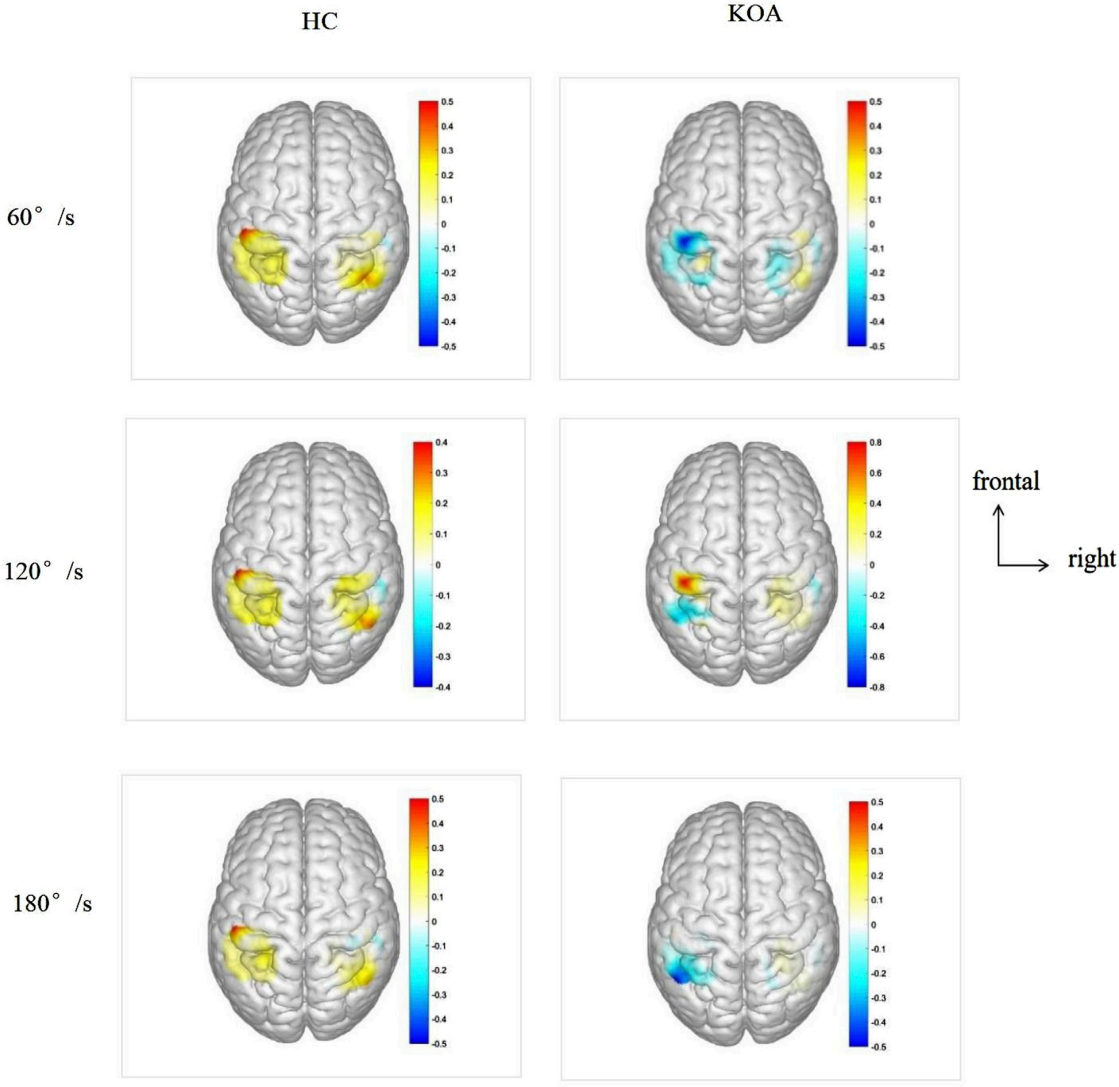
Cerebral cortex activation maps show mean HbO levels in HC and KOA patients during unilateral knee isokinetic movements at three speeds. The transition from blue to red signifies increasing activation intensity from low to high. HbO, Oxyhemoglobin; KOA, knee osteoarthritis; HC, healthy controls.

Figure 3 cerebral cortex activation maps show mean HbO levels in HC and KOA patients during unilateral knee isokinetic movements at three speeds. For HC, bilateral activation of M1, S1, and SAC was observed across all speeds. There was no statistically significant difference in activation levels between the left and right of cerebral cortex, except for S1 under 180°/s speed, but the contralateral activation was dominant, showing the phenomenon of high contralateral activation. For KOA patients, there were no statistical difference in the activation level between the left and right of the cerebral cortex, with both sides showing lower activation levels compared to HC. As shown in Table 4.

**TABLE 4:** Bilateral differences in cerebral cortex activation in HC and KOA patients.

		HC			KOA		
		60°/s	120°/s	180°/s	60°/s	120°/s	180°/s
M1	L	0.52 ± 1.60	0.32 ± 1.18	0.53 ± 1.03	0.01 ± 0.37	0.00 ± 0.22	0.04 ± 0.24
	R	0.14 ± 0.50	0.19 ± 0.43	0.06 ± 0.32	0.08 ± 0.45	0.15 ± 0.42	0.02 ± 0.28
S1	L	0.47 ± 1.08	0.33 ± 0.61	0.40 ± 0.89^a^	0.21 ± 1.07	0.09 ± 0.63	0.11 ± 0.67
	R	0.16 ± 0.71	0.15 ± 0.56	0.03 ± 0.76	0.05 ± 0.69	0.11 ± 0.41	0.04 ± 0.37
SAC	L	0.52 ± 0.83	0.37 ± 0.50	0.43 ± 0.89	0.25 ± 0.78	1.04 ± 3.64	1.07 ± 2.47
	R	0.32 ± 1.57	0.19 ± 1.12	0.22 ± 1.08	0.26 ± 0.89	0.04 ± 0.91	0.06 ± 1.34

^a^ : statistically significant, *P* < 0.05.

KOA, knee osteoarthritis; HC, healthy controls; M1, primary motor; S1, primary somatosensory; SAC, somatosensory association cortex.

### 3.4 Differences in cerebral cortical activation between KOA patients and HC

**FIGURE 4:**
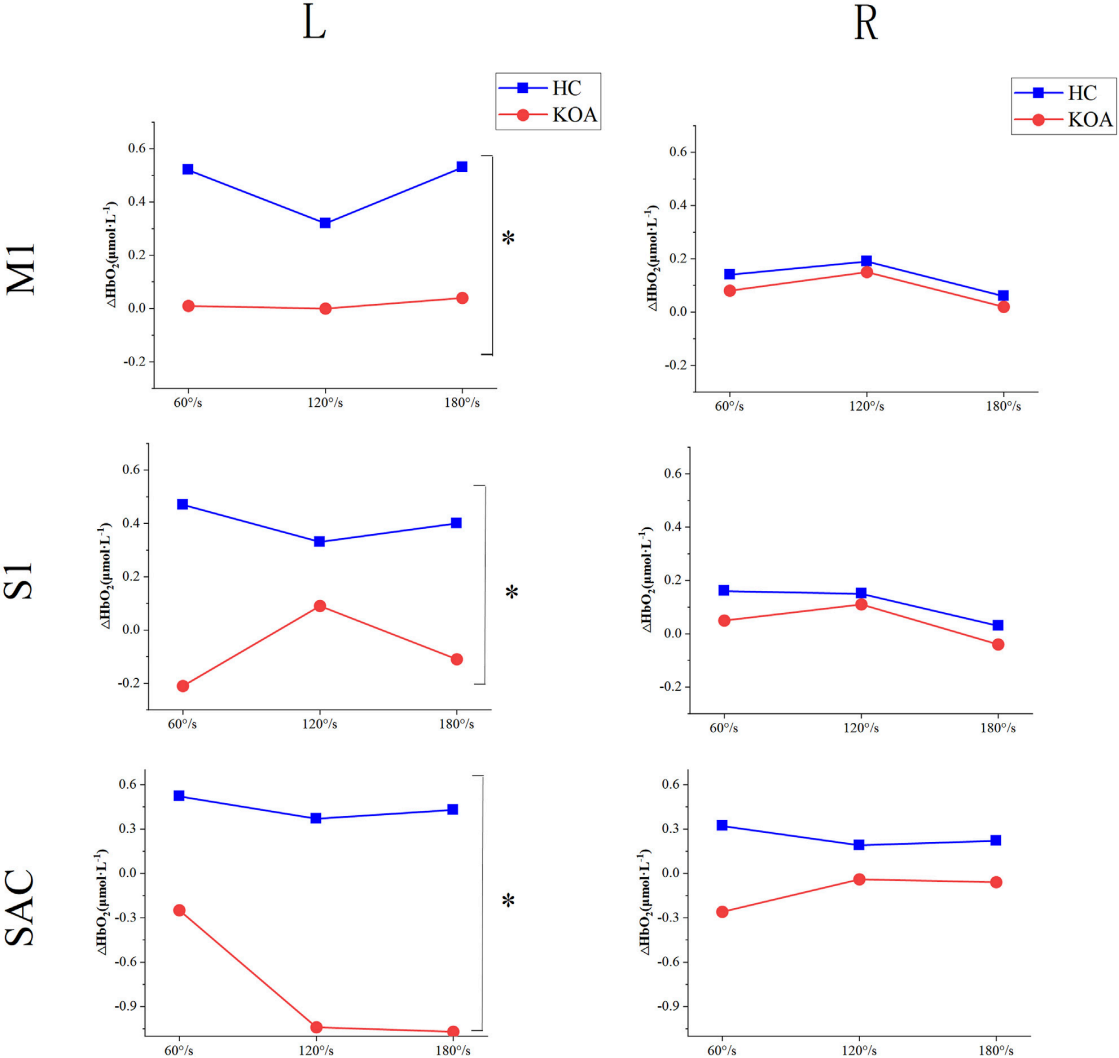
Differences in cerebral cortical activation between KOA patients and HC. KOA, knee osteoarthritis; HC, healthy controls; S1, primary somatosensory; M1, primary motor; SAC, somatosensory association cortex.

Figure 4 shown the differences in cerebral cortical activation between KOA patients and HC. For the left of M1, S1, and SAC, there was no significant interaction effect between group and speed. Similarly, there was no significant interaction effect on the right. A significant main effect of group was found for the left of M1, S1, and SAC (*P*_M1_ = 0.017; *P*_S1_ = 0.005; *P*_SAC_ = 0.002), while the right side had no difference. The left of M1, S1, and SAC in KOA patients were significantly lower than those in HC, indicating that the left side of cerebral cortex were inhibited in KOA patients compared with that in HC. Additionally, speed had no significant main effect on either left or right side.

### 3.5 Correlations between cerebral cortical activation and clinical pain (VAS) and KOA functional symptoms (WOMAC) measures

**FIGURE 5:**
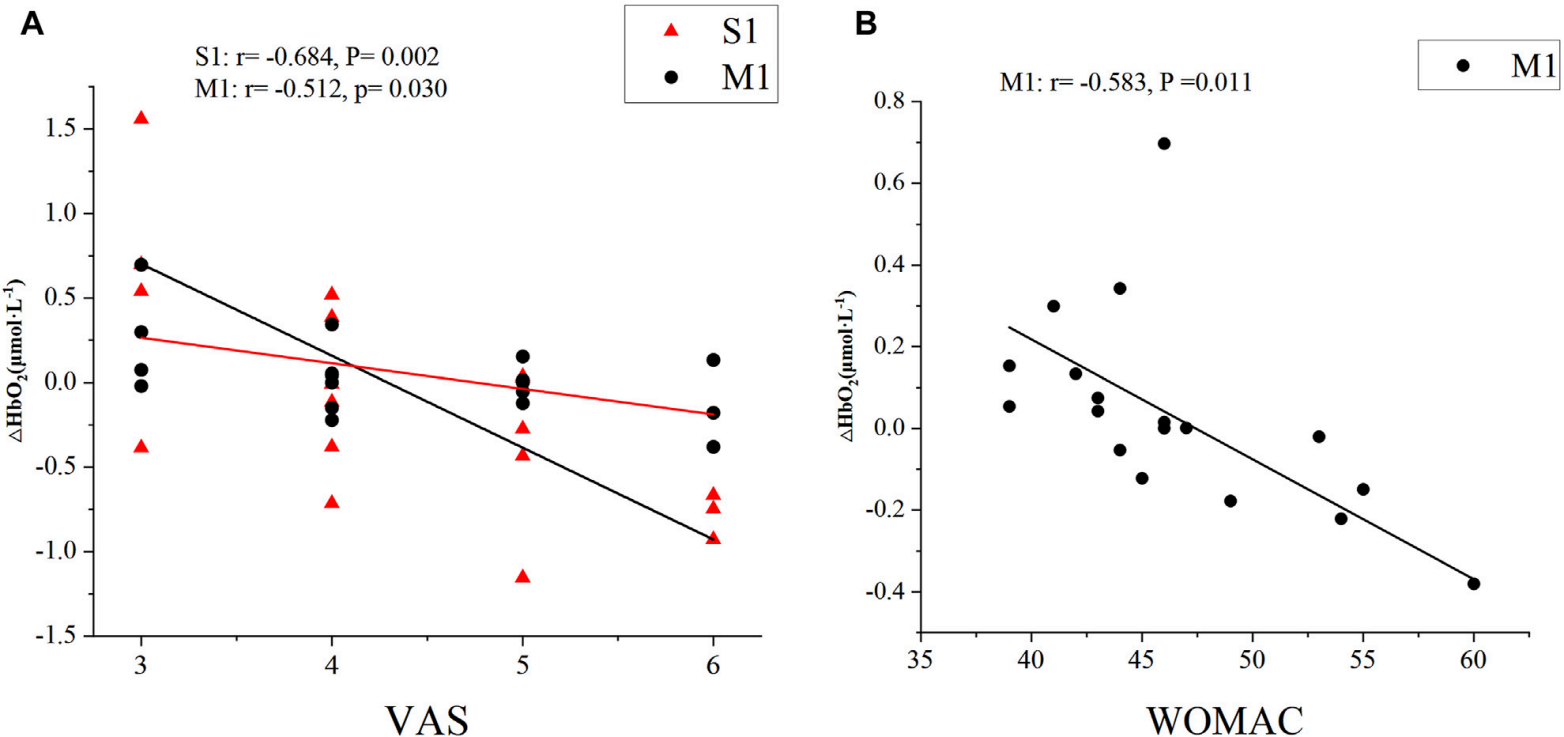
Correlations between cerebral cortical activation and **(A)** VAS, **(B)** WOMAC scores. VAS, Visual Analog Scale; WOMAC, Western Ontario and McMaster Universities Osteoarthritis Index; S1, primary somatosensory; M1, primary motor.

For the clinical pain, correlation analysis revealed that the VAS scores in KOA patients were significantly negatively correlated with the left M1 (moderate correlation: *r* = −0.512, *P* = 0.030) and S1 (high correlation: *r* = −0.684, *P* = 0.002) values under isokinetic movement at 180°/s speed (Figure 5A). For the KOA functional symptoms, correlation analysis revealed that the WOMAC scores in KOA patients were significantly negatively correlated with the left M1 (moderate correlation: *r* = −0.583, *P* = 0.011) values under isokinetic movement at 180°/s speed (Figure 5B). In addition, there was no correlation between VAS and WOMAC and SAC.

## 4 Discussion

The main objective of this study was to investigate the alterations in brain activation patterns in KOA patients during movement. Specifically, we compared the changes of cerebral cortical blood flow monitored by fNIRS between KOA patients and healthy subjects during isokinetic movement at different velocities. The correlation between changes in brain activation and pain severity and dysfunction in KOA patients were also evaluated to provide evidence and potential targets for brain activation changes for subsequent interventions, and open a new perspective for the rehabilitation of musculoskeletal diseases.

The results of peak torque showed that KOA patients exhibited significantly decreased muscle strength during isokinetic movements. This is consistent with previous findings that osteoarthritis patients exhibit decreased muscle strength and muscle imbalance (Neil et al., 2010; Pua et al., 2011). This may be due to the pathological changes of limited joint mobility and muscle function deterioration in osteoarthritis patients (Hideki et al., 2011). The limited joint mobility can reduce the range of motion of the muscles around the joint, thereby limiting the muscle’s ability to contract and unable to exert maximum force. Meanwhile, the chronic inflammatory response caused by osteoarthritis can also lead to muscle atrophy and dysfunction, further reducing the patient’s muscle strength level (Zhang et al., 2024). In addition, symptoms such as joint pain and muscle spasms may also inhibit the patient from exerting maximum muscle strength during the test. Therefore, the combined effects of limited joint mobility, muscle dysfunction, and pain lead to lower peak torque values in osteoarthritis patients compared to healthy individuals during isokinetic muscle strength testing. This provides a basis for developing targeted muscle strength training in clinical practice.

In this study, we used the fNIRS method as a neuroimaging tool for brain activation changes during movement. fNIRS has been recognized as a reliable and effective alternative to fMRI and EEG measures in previous research (Xu et al., 2010; Antonio et al., 2017). This method is a novel noninvasive technique that boasts attributes such as safety, portability, and the ability to tolerate motion artifacts, which enhance the methodological robustness and strength of studies investigating real-time brain networks during movement (Cristina et al., 2020; Alka et al., 2021).

Throughout the experimental activities, we observed that healthy people showed a large increase in cortical blood flow in the contralateral brain region during unilateral knee movement, including M1, S1and SAC. Many research had provided evidence for the lateralization of motor and somatosensory functions in the brain (Kwong et al., 1992; Jonathan P et al., 2010; Alahmadi et al., 2015). For example, Kwong and Jonathan et al. used fMRI technique to find that the contralateral motor cortex was significantly activated when subjects performed unilateral limb movements (Kwong et al., 1992; Jonathan P et al., 2010). Alahmadi et al. (2015) reported differences in activation changes of brain regions when using dominant versus non-dominant hands during a motor task, reflecting the lateralized characteristics of motor functions. Additionally, several studies have investigated the cortical activation patterns in response to unilateral limb stimulation, and found that single-sided limb stimulation led to significant activation in the contralateral cortical regions, supporting the lateralized nature of somatosensory processing (Bong Soo et al., 2003; Susan et al., 2008; Simone et al., 2010). Similarly, Baumgärtner et al. (2010) employed high-resolution fMRI and detected obvious somatotopic representations of heat and mechanical pain in the operculo-insular cortex, further demonstrating the lateralized organization of pain-related functions. Taken together, the results of this study are consistent with the above research, which proves that unilateral limb movement or sensory stimulation can cause the activation of the contralateral cortex, reflecting the anatomical and functional lateralization of motor and somatosensory functions.

However, in some disease states, such as osteoarthritis and stroke, activation of the cerebral cortex may be inhibited. In this study, it was found that compared with healthy subjects, the activation of the contralateral brain region in KOA patients was remarkable inhibited during limb movement. This phenomenon is also manifested in other diseases. For instance, Li et al. found that compared to healthy controls, patients with chronic ankle instability showed significantly decreased activation intensity of the motor cortex during unilateral movements (Li et al., 2024). Similarly, Hu et al. (2017) reported that stroke patients exhibited lower cortical activation levels than healthy individuals during movements. Furthermore, Karin et al. (2012) demonstrated that chronic pain patients also displayed reduced cortical activation during limb movements relative to healthy controls. These findings suggest that disease states may alter the patterns of cortical activation during movement, which may be related to impaired sensorimotor function in patients.

In addition, we observed that the activation of the cerebral cortex was not affected by the speed environment during limb movement at all speeds. This may be because under isokinetic muscle strength movement, we find that although the motion speed was different under different conditions, the torque force of the patient remains unchanged. Numerous studies have utilized fMRI and fNIRS to elucidate the relationship between brain activity in the motor regions and the levels of force exerted (Kenichi et al., 2014; Teo et al., 2023). It has been reported that the activation of contralateral M1 increases logarithmically with the increase of force output, which is inconsistent with the observed motion behavior (Karin B et al., 2012). Additionally, considering the presence of cortico-cortical connections from the sensory cortex to the motor cortex, it is reasonable to infer that information from S1 was involved in the control of movement, either through direct or indirect means. It is worth noting that the S1 region showed activation during movement in this study.

At the same time, we found that the clinical pain and dysfunction in KOA patients were significantly negatively correlated with activation levels of specific brain regions under isokinetic movement at 180°/s speed. Existing studies have shown that chronic pain can cause changes in the functional activity of the cerebral cortex, such as decreased activation in the sensory cortex, motor cortex, and prefrontal cortex, which may reflect the brain’s adaptive regulatory mechanism to persistent pain stimuli (Apkarian et al., 2011; Baliki et al., 2012). The results of this study further confirm this phenomenon, suggesting that the functional changes in the cerebral cortex of knee osteoarthritis patients may be the neurophysiological basis of their pain and functional impairment. This negative correlation may be due to the neuroplastic changes in the brain in response to chronic pain, including inhibition of sensory pathways, impairment of motor control, and imbalance of emotional regulation (Seminowicz et al., 2013). In addition, some studies have also found that through neuromodulation techniques such as tDCS, the activity of the cerebral cortex can be regulated, thereby improving the symptoms of patients with chronic pain (Khedr et al., 2005; Pollonini et al., 2020; Montero-Hernandez et al., 2023). These studies support the findings of this study, that is, the activation level of the cerebral cortex is closely related to pain perception. On the other hand, these showed correlations at speed of 180°/s, which may be mainly due to the low external interference at high speed and higher consistency of brain activation among patients. In summary, these research results provide important evidence for further exploring the neural mechanisms of pain and developing new treatment methods.

There are several limitations in this study that need to be considered. First, we only recruited right-sided KOA patients, so it is difficult to directly extrapolate results to left-sided or bilateral KOA patients. Second, we only performed simple unilateral limb movements, so we cannot know the effects of complex multi-limb movements on brain activation. Finally, the current study did not perform traditional neuroimaging (such as fMRI) assessments, limiting further understanding of the potential mechanisms that might explain the under-activation observed in patients with KOA. Further studies may address these shortcomings in the future by expanding the inclusion criteria of patients, conducting complex movement studies of multiple limbs, and using structural neuroimaging.

## 5 Conclusion

In conclusion, this study further clarified that there was high contralateral activation of the sensorimotor cortex during unilateral knee movement in healthy individuals, whereas contralateral cortical activation was suppressed in KOA patients. Furthermore, the clinical pain of KOA patients was negatively correlated with the activation level of the contralateral S1 region, and the dysfunction was negatively correlated with the activation level of the contralateral S1 and M1 regions. These findings can provide a better understanding of KOA brain science and are expected to contribute to the development of central intervention for the disease.

## Data Availability

The raw data supporting the conclusions of this article will be made available by the authors, without undue reservation.
